# Altered expression of genes involved in ganglioside biosynthesis in substantia nigra neurons in Parkinson’s disease

**DOI:** 10.1371/journal.pone.0199189

**Published:** 2018-06-14

**Authors:** Jay S. Schneider

**Affiliations:** Department of Pathology, Anatomy and Cell Biology, Thomas Jefferson University, Philadelphia, Pennsylvania, United States of America; Central Michigan University, UNITED STATES

## Abstract

Reduced expression of GM1 and other major brain gangliosides GD1a, GD1b and GT1b have been reported in Parkinson’s disease (PD) brain. Mechanisms underlying these changes are unclear but may be due to a deficit in the ganglioside biosynthetic process. The present study examined the extent to which deficits in gene expression of key biosynthetic enzymes involved in synthesis of GM1 and GD1b (B3galt4) and GD1a and GT1b (St3gal2) exist in neuromelanin-containing neurons in the PD substantia nigra (SN). In situ hybridization histochemistry was used to examine gene expression of *B3GALT4* and *ST3GAL2* in neuromelanin-containing neurons in the SN in 8 normal controls (61–92 yrs.) and 7 PD subjects (77–95 yrs). There was a significant decrease in both *B3GALT4* and *ST3GAL2* gene expression in residual neuromelanin-containing cells in the SN of PD patients compared to age-matched neurologically normal controls. These changes appeared to be cell-type specific as abundant *B3GALT4* and *ST3GAL2* gene expression was observed in non-neuromelanin containing neurons located outside of the SN in the PD brain. These data show that residual neuromelanin-containing neurons in the PD SN have decreased expression of the ganglioside biosynthetic genes *B3GALT4* and *ST3GAL2*, consistent with previous reports of decreased levels of gangliosides GM1, GD1a, GD1b and GT1b in the PD SN. These changes may increase the vulnerability of these neurons to degeneration in response to a variety of potential stressors.

## Introduction

Parkinson’s disease (PD) is a progressive disorder primarily characterized by degeneration of substantia nigra (SN) dopaminergic neurons with associated loss of dopamine (DA) in the striatum (caudate nucleus and putamen), and accumulation of insoluble protein aggregates (in Lewy bodies and Lewy neurites), with α-synuclein being the most abundant component [1]. The mechanisms contributing to the onset and progression of PD are not completely known, but are hypothesized to involve dysfunction of mitochondria, increased oxidative stress resulting in multiple forms of oxidative damage, and defects in the ubiquitin-proteasome system and lysosomal pathways (leading to an increase in and accumulation of misfolded proteins) [2, 3].

In addition to the potential disease mechanisms listed above, there has also been recent interest in the role of lipids in neurodegenerative diseases and in particular, in the pathogenesis of PD. Abnormalities in the content and composition of various lipids have been reported in PD brain [ex.,[4–6]]. A recent plasma metabolic profiling study suggested that abnormal metabolic changes in PD plasma were mainly associated with mitochondrial function and lipid metabolism [7]. Additionally, mutations in the beta-glucosidase gene (GBA) coding for glucocerebrosidase, which breaks down glucosylceramide into glucose and ceramide, have been suggested to be an important risk factor for development of sporadic PD [8].

**Fig 1:**
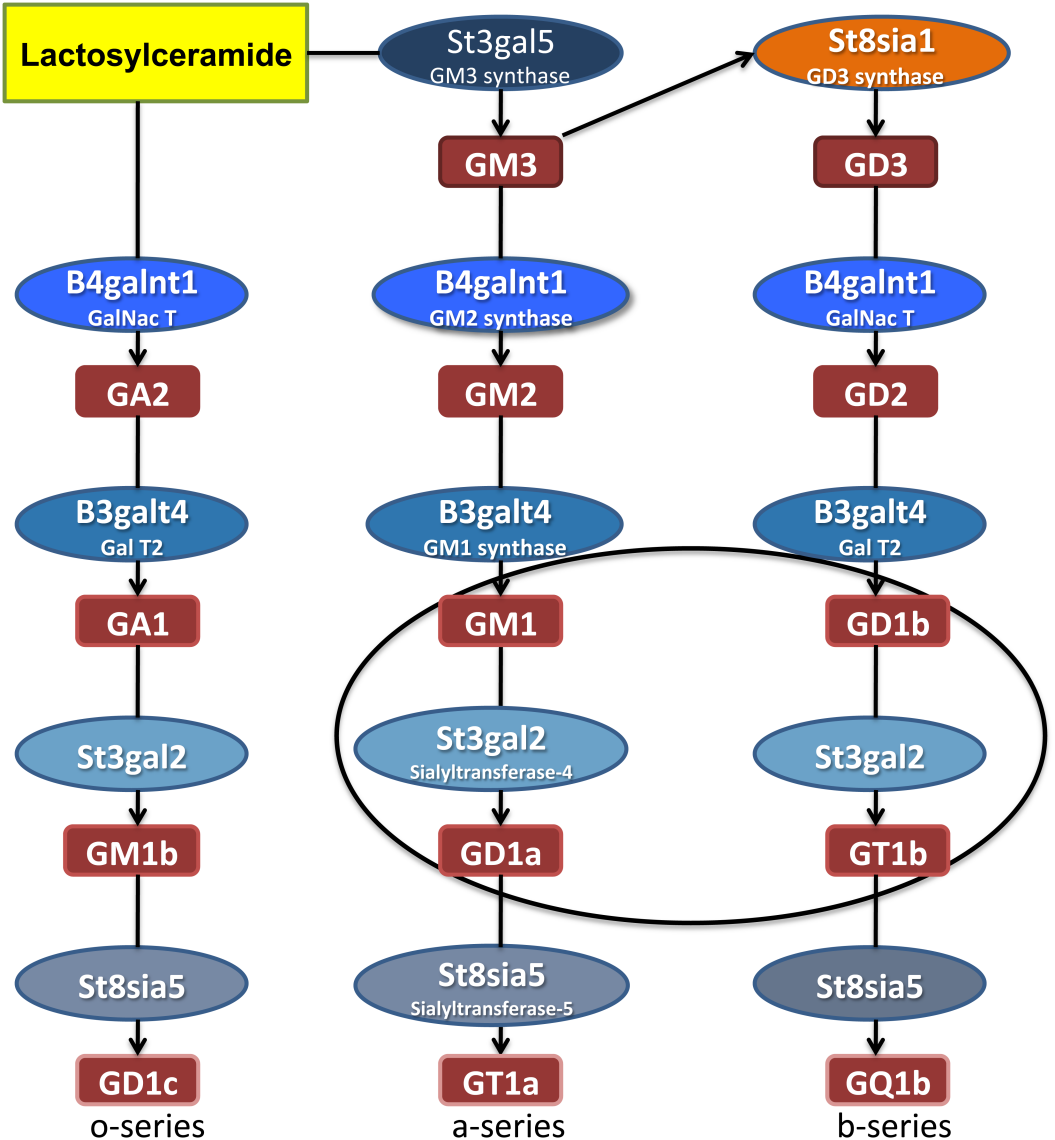
Ganglioside biosynthetic pathways. The main gangliosides found in brain are a-series and b-series gangliosides (encircled). Gangliosides are synthesized by sequential addition of sugars and sialic acid residues onto a sphingosine backbone through the action of various glycosyltransferases (ex., St3gal5, B4galnt1, B3galt4, St8sia1) and sialyltransferases (St3gal2, St8sia5).

Of particular interest and functional significance are reported changes in gangliosides in PD brain, due at least in part to their role in lipid raft structure/function and numerous cell signaling mechanisms, [9–12]. Gangliosides are sialic acid containing glycosphingolipids, of which the major species in brain are a- and b-series gangliosides GM1, GD1a, GD1b, and GT1b [13]. The monosialoganglioside GM1 is of particular interest as it exerts neurotrophic or neuroprotective effects under a variety of circumstances and influences numerous cellular activities at the level of plasma and intracellular membranes, where it influences diverse cellular functions including Ca^2+^ homeostasis, mitochondrial function, and lysosomal integrity, among other processes [14–17]. Reduced numbers of GM1 ganglioside-containing cells were reported in SN pars compacta neurons in brains from PD patients, using FITC-cholera toxin B histochemistry: only approximately 20% of dopamine neurons in the PD SN expressed GM1 compared to almost 62% in non-PD controls [18]. Reduced expression of GM1, GD1a, and GD1b were reported in the occipital cortex of PD patients using HPTLC [19]. Using HPTLC, we have also recently described significant decreases in GD1a, GD1b, and GT1b in SN from male PD patients, along with a smaller magnitude decrease in GM1 that did not reach statistical significance [20]. In contrast, we found no significant changes in levels of GM3, GM2, or GD3 in PD SN. This led us to hypothesize that a potential problem in ganglioside biosynthesis may exist in the PD SN downstream from GM3, GM2 and GD3 and that a potential change in expression of key biosynthetic enzymes involved in synthesis of GM1 and GD1b (B3galt4) and GD1a and GT1b (St3gal2) may exist (the main ganglioside biosynthetic pathways in brain are shown in Fig 1). Since ganglioside expression is cell type-specific [21], interpretation of potential gene expression changes such as these in SN homogenates from PD brain could be complicated due to loss of SN neurons, glial responses, and signals from white matter, the current study was performed to examine *B3GALT4* and *ST3GAL2* gene expression in neuromelanin-containing neurons in the SN of PD patients and non-PD controls using in situ hybridization histochemistry.

## Materials and methods

### Brain tissues and sectioning

Fully anonymized human brain tissue was obtained through the NIH NeuroBioBank and sourced from the NICHD Brain and Tissue Bank for Developmental Disorders at the University of Maryland, Baltimore, MD, the Harvard Brain Tissue Resource Center, Belmont, MA, which is supported in part by HHSN-271-2013-00030C, and from the Human Brain and Spinal Fluid Resource Center, VA West Los Angeles Healthcare Center, 11301 Wilshire Blvd., Los Angeles, CA, which is sponsored by NINDS/NIMH, the National Multiple Sclerosis Society, and the Dept. of Veterans Affairs. The clinical diagnosis of Parkinson’s disease was confirmed at autopsy by presence of gross depigmentation of the SN, microscopic confirmation of SN cell loss and/or presence of Lewy bodies in the SN, and normal findings in other brain regions sampled. Frozen tissue blocks containing the SN were stored at -80°C, warmed to -20°C in a cryostat, embedded in optimal cutting temperature (OCT) compound, sectioned at 14 μm thickness and mounted (2 sections/slide) onto Fisherbrand^™^ Superfrost^™^ Plus microscope slides. Slides were kept in the cryostat at -20°C for 1 hr. to dry, placed in cold slide boxes, and stored at -80°C in vacuum-sealed bags until used.

### In situ hybridization

In situ hybridization was performed using the RNAscope 2.5 HD Chromogenic Assay kit (Advanced Cell Diagnostics, Inc.) using a protocol, with slight modifications, as recommended in the RNAscope 2.5 HD Chromogenic Assay user manual for fresh frozen tissue. Briefly, slides were removed from the -80°C freezer and immediately placed in glass Coplin jars containing ice cold 4% paraformaldehyde in PBS and fixed for 2 hrs. at 4°C. Slides were removed from the 4% paraformaldehyde and placed in 50% EtOH for 5 min. at room temperature, followed by 70% EtOH for 5 min. at room temperature, and then 100% EtOH for 5 min. at room temperature. Slides were then stored in 100% EtOH overnight at -30°C. The following day, slides were removed from the 100% EtOH and allowed to air dry for at least 15 min. A hydrophobic barrier was then drawn around each section (Immedge^™^ pen) and sections were incubated in hydrogen peroxide for 10 min at room temperature, rinsed in dH_2_0, incubated in RNAscope Protease IV for 30 min at room temperature, and rinsed in PBS. One section on each slide was then incubated in either a custom human gene-specific RNAscope probe for *B3GALT4* or human *ST3GAL2* and the other section was incubated in a positive control probe (human Cyclophilin B (*PPIB*)). Some additional slides were also incubated with a negative control probe (bacterial *dapB*) to verify the specificity of the assay. Slides were place in a sealed, humidified tray and incubated for 2 hrs. at 40°C. After removal of the probes and washes in RNAscope wash buffer, the sections were sequentially hybridized to a cascade of amplification molecules, culminating in binding to HRP-labeled probes, followed by signal detection with Fast Red substrate (RNAscope 2.5 HD Detection Kit-Red, ACB Bio) to detect target RNA. Slides were then counterstained with Hematoxylin, dried overnight and coverslipped.

### Data analysis

All slides were initially evaluated for positive control signal strength at 20x magnification. If positive control sections had signal of at least 20–30 dots/cell (each dot represents a single RNA molecule), the tissue was considered to contain RNA of suitable integrity for analysis of the expression of the target genes. For the purposes of this study, if a dot cluster was visible, it was counted as one dot. For each gene of interest, the number of dots/cell was counted on coded slides (to blind the observer to disease status (PD or Control) of the sample) at 20x, with a range of 20–55 and 20–45 cells analyzed for *B3GALT4* expression in control and PD cases, respectively, and a range of 18–20 and 18–34 cells analyzed for *ST3GAL2* expression in control and PD cases, respectively. To be counted, a cell needed to have an intact hematoxylin-positive cell body and neuromelanin clearly visible. All dots on a cell were counted; there were no inclusion or exclusion criteria for selecting a dot to be counted or not counted. Dot count data from normal control cases and PD cases were then compared using unpaired Student’s t test.

## Results

There were no significant differences between the ages or post-mortem intervals (PMI) between the neurologically normal control subjects and PD subjects (Table 1). All sections analyzed were from cases with positive control (*PPIB*) signal of at least 20–30 dots/cell and with presence of dot clusters, signifying good integrity RNA. Analyses of negative control (*dapB*) sections showed no signal present and verified lack of non-specific signal in the assay (S1 Fig).

**Table 1:** Subject characteristics.

**Control**	**Age (yrs)**	**PMI (hrs)**	**Sex**
1	78	5	M
2	76	16	M
3	78	21	M
4	71	17	M
5	63	23	F
6	88	16	M
7	82	14	M
8	92	15	M
Mean	78.5 ± 3.2	15.9 ± 1.9	
**PD**	**Age (yrs)**	**PMI (hrs)**	**Sex**
1	95	10	M
2	75	5.5	M
3	76	15	M
4	88	21	M
5	77	10	M
6	84	6	F
7	77	22	M
Mean	81.7 ± 2.9	12.8 ± 2.6	

PMI = post-mortem interval

**Fig 2:**
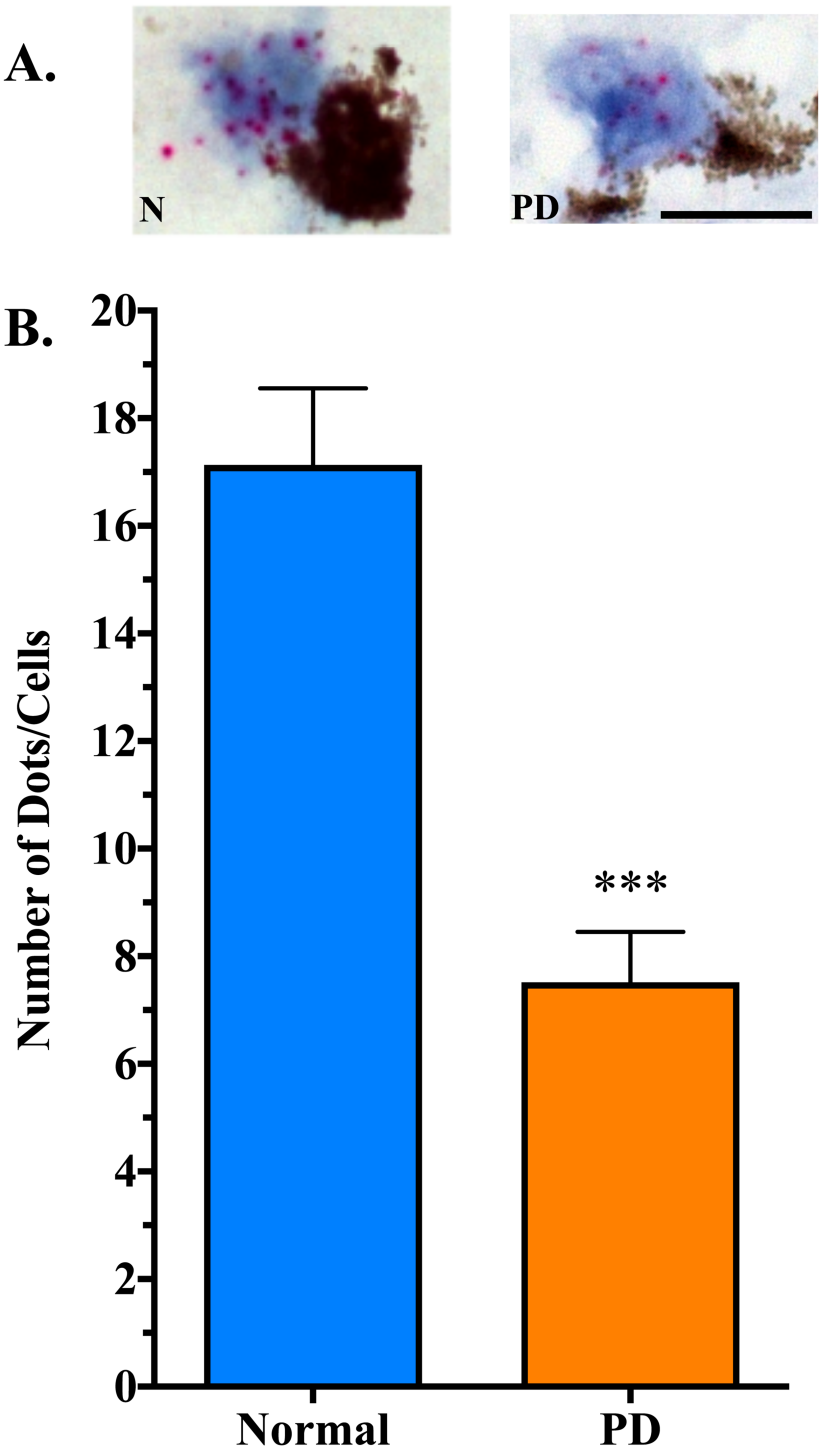
*B3GALT4* gene expression is lower in residual neuromelanin-containing cells in the PD SN than in control SN neurons. A. In situ hybridization for *B3GALT4* gene expression in control (N) and PD substantia nigra (SN). Using the RNAscope 2.5 HD Detection Kit-Red (ACB Bio), each red dot represents a single RNA molecule. Scale = 25 μm. B. Counts of the number of dots (RNA molecules) per neuron showed significantly less *B3GALT4* RNA in cells in the PD SN (196 cells analyzed) compared to the control SN (269 cells analyzed). ***P = 0.0001 compared to Normal. Neuromelanin appears as brown/black aggregates in cells.

**Fig 3:**
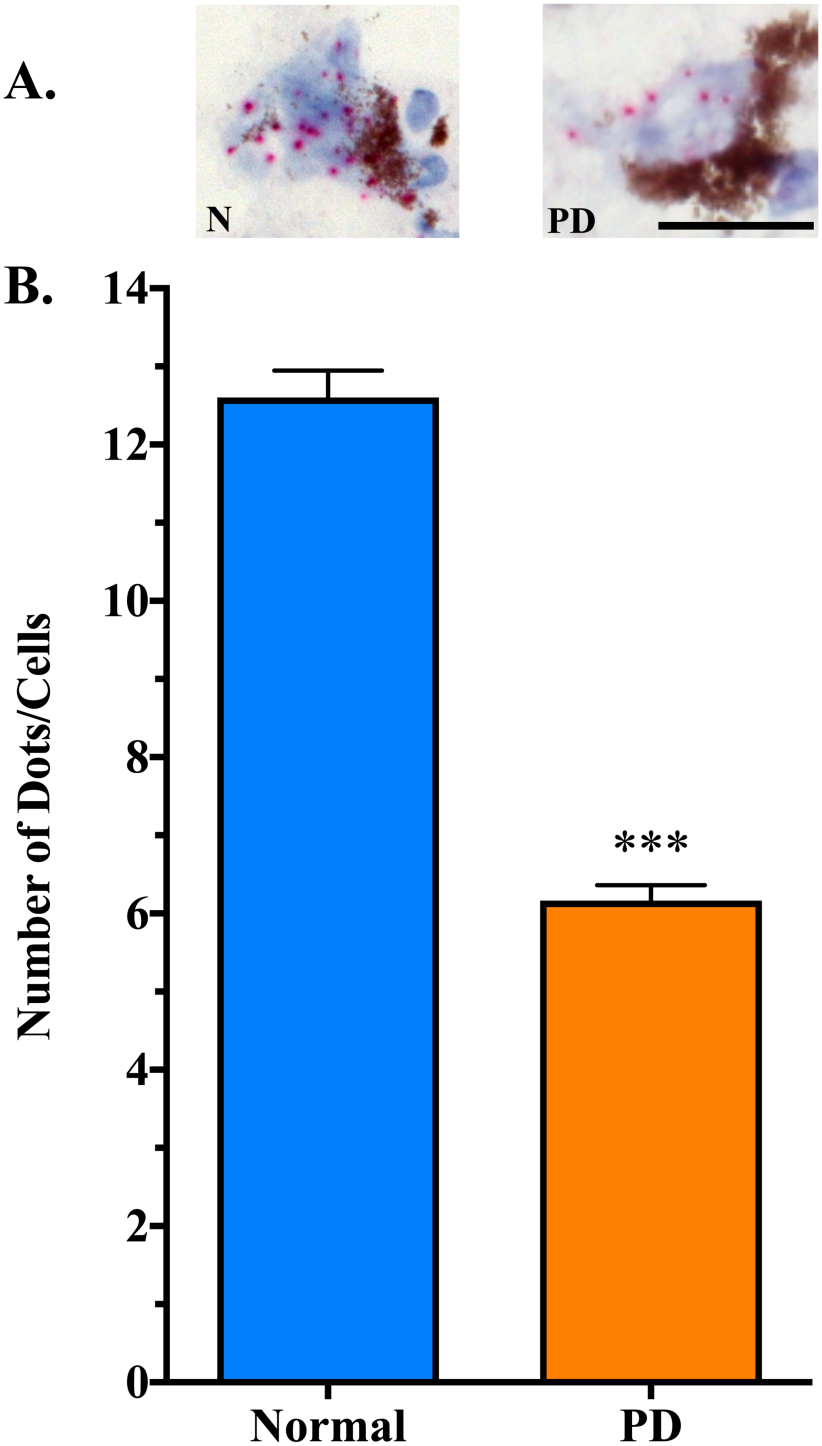
*ST3GAL2* gene expression is lower in residual neuromelanin-containing cells in the PD SN than in control SN neurons. A. In situ hybridization for *ST3GAL2* gene expression in control (N) and PD substantia nigra (SN). Using the RNAscope 2.5 HD Detection Kit-Red (ACB Bio), each red dot represents a single RNA molecule. Scale = 25 μm. B. Counts of the number of dots (RNA molecules) per neuron showed significantly less *ST3GAL2* RNA in cells in the PD SN (218 cells analyzed) compared to the control SN (146 cells analyzed). ***P < 0.0001 compared to Normal. Neuromelanin appears as brown/black aggregates in cells.

**Fig 4:**
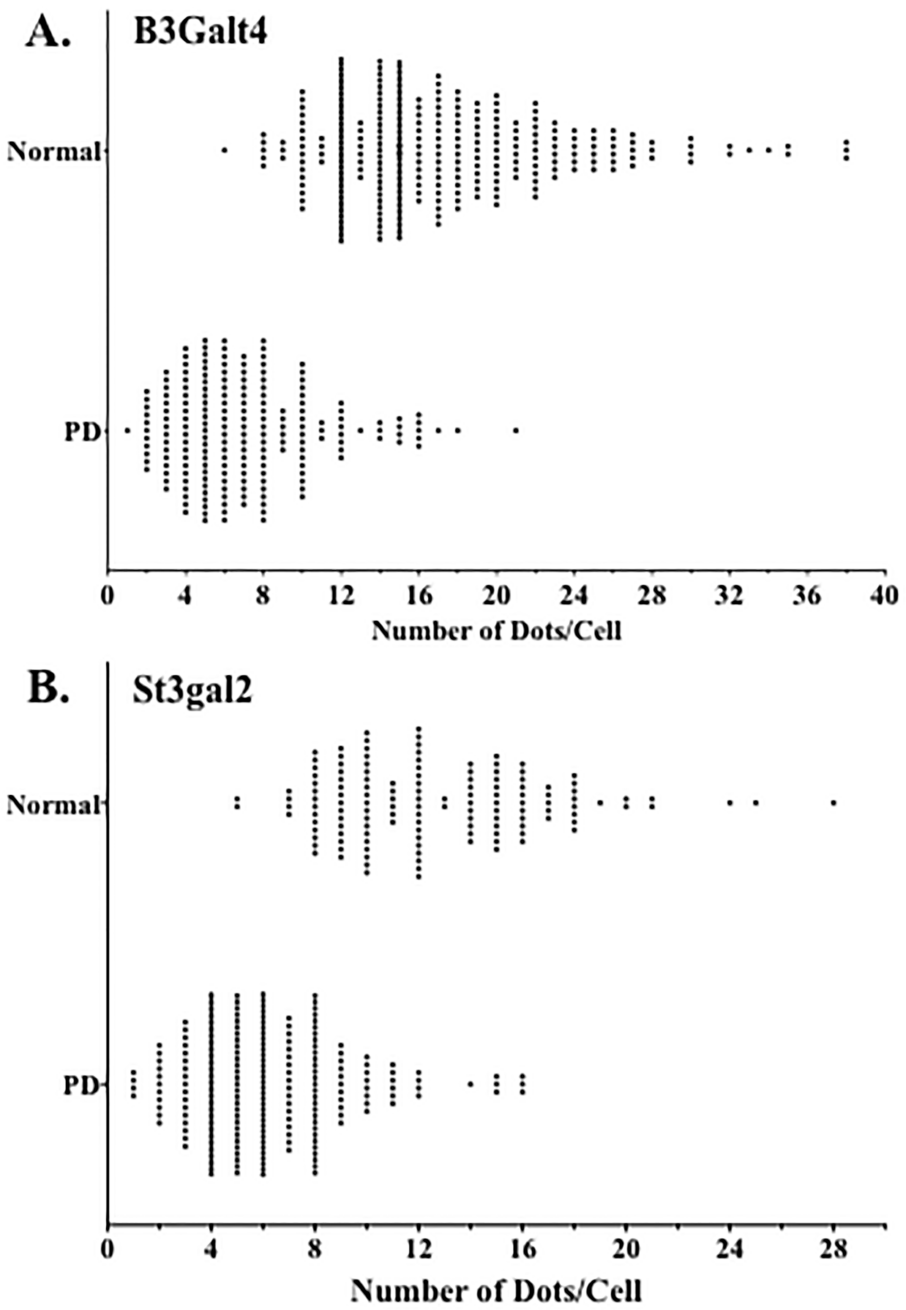
Graphs showing the distribution *B3GALT4* (a) and *ST3GAL2* (B) gene expression per neuromelanin-containing neuron in normal and PD substantia nigra (SN). Each dot represents the number of RNA molecules (red dots counted per cell) counted in each cell sampled. For both genes, there is overlap in the extent to which these genes are expressed in normal and PD SN neurons.

**Fig 5:**
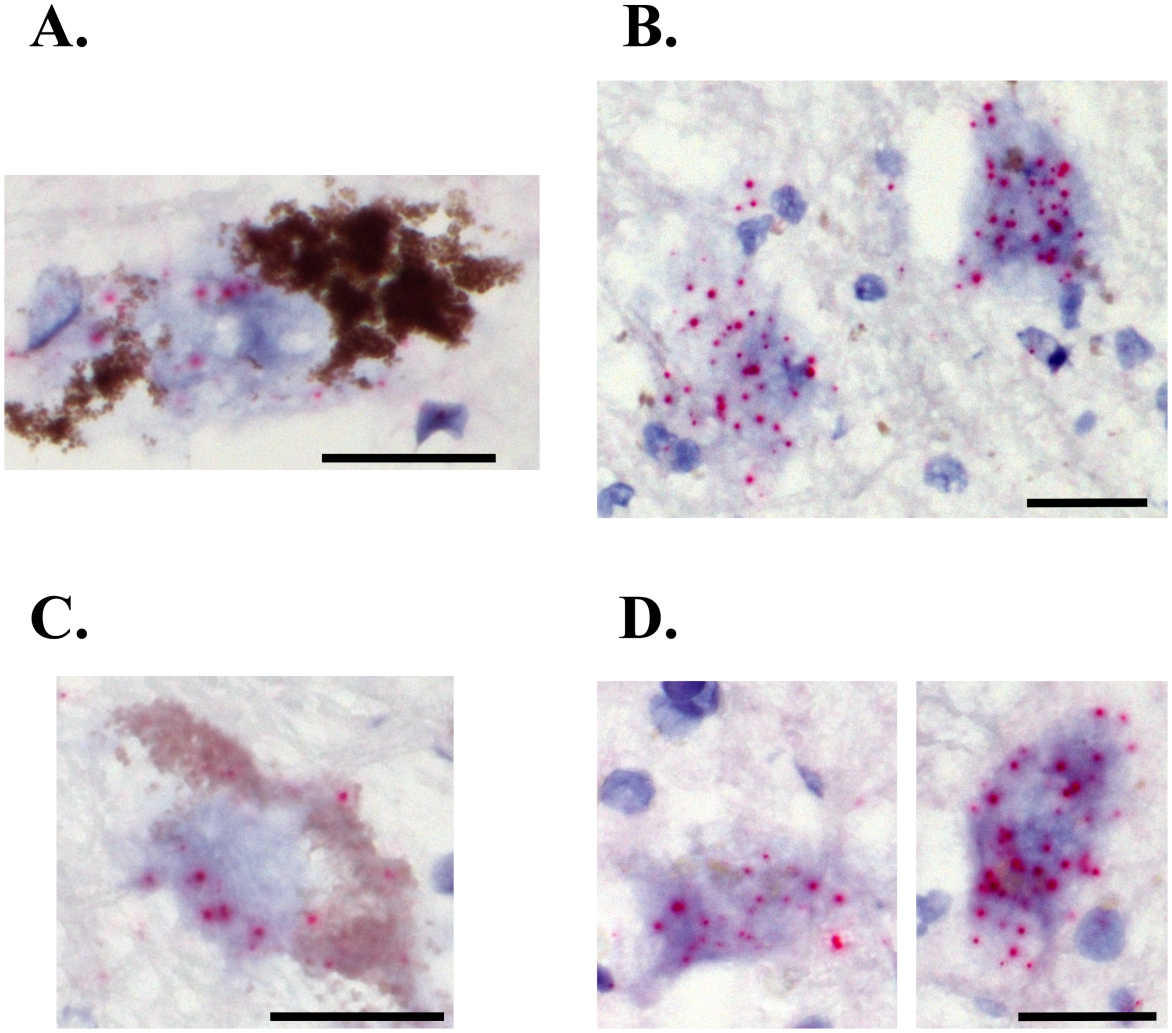
Within a section, some cells located outside of the substantia nigra (SN) showed much higher *ST3GAL2* and *B3GALT4* gene expression levels than neuromelanin-containing cells located in the SN in the PD brain. A. *ST3GAL2* gene expression was relatively low in neuromelanin-containing cells in the PD SN (as indicated by the low number of red dots in cells containing brown/black aggregates indicative of neuromelanin) compared to the level of *ST3GAL2* gene expression that could be observed in neurons in the same section located outside of the SN (B). Similarly, *B3GALT4* gene expression was relatively low in neuromelanin-containing cells in the PD SN (C, as indicated by the low number of red dots in cells containing brown/black aggregates indicative of neuromelanin) compared to the level of *B3GALT4* gene expression that could be observed in neurons in the same section located outside of the SN (D). Scale = 25 μm for each.

There was a significant decrease in *B3GALT4* expression in residual neuromelanin-containing cells in the SN of PD patients (7.5 ± 0.9 dots/cell, n = 7) compared to neurologically normal controls (17.1 ± 1.4 dots/cell, n = 8) (t = 5.293, df = 14, P = 0.0001) (Fig 2A and 2B). Similar to what was observed for *B3GALT4* expression, there was a significant decrease in *ST3GAL2* expression in residual neuromelanin-containing cells in the SN of PD patients (6.0 ± 0.4 dots/cell, n = 7) compared to neurologically normal controls (12.5 ± 1.1 dots/cell, n = 8) (t = 5.532, df = 14, P < 0.0001) (Fig 3A and 3B). Scatter plots of these data show some overlap in expression between control and PD groups, with approximately 39% of neuromelanin-containing neurons in the PD SN within the lower end of the normal range of *B3GALT4* expression (Fig 4A). Scatter plots also show some overlap in *ST3GAL2* expression between the two groups, with approximately 50% of neuromelanin-containing neurons in the PD SN showing *ST3GAL2* expression levels within the lower end of the normal range of *ST3GAL2* expression (Fig 4B). The decrease in expression of *ST3GAL2* and *B3GALT4* in residual dopaminergic neurons in the PD cases appeared to be specific for this cell type. Abundant *ST3GAL2* and *B3GALT4* gene expression was observed in some non-dopaminergic neurons located outside of the SN in the PD brain, in the same sections in which decreased gene expression was observed in the SN (Fig 5).

## Discussion

The results reported here show for the first time, decreases in gene expression of the glycosyltransferase *B3GALT4* (GM1/GD1b synthase) and the sialyltransferase *ST3GAL2* in neuromelanin-containing neurons in the PD SN compared to non-PD controls. These results suggest a problem with the ganglioside biosynthetic machinery in remaining dopaminergic neurons in the PD brain and extend previous findings showing decreased numbers of GM1 ganglioside-expressing cells in the PD SN [18] and decreased levels of the major brain gangliosides, GM1, GD1a, GD1b and GT1b in whole SN homogenates from PD patients [20]. Thus, the decreased expression of *B3GALT4* and *ST3GAL2* mRNA likely directly underlies the decreased expression of GM1, GD1a, GD1b and GQ1b observed in PD SN [20], potentially contributing to the vulnerability of these neurons to neurodegeneration. Since gangliosides are expressed in a cell-specific manner [21], it is important to study expression of gangliosides and their biosynthetic processes in specific cells of interest in normal brain and in different disease states in order to gain more insight into the role that changes in ganglioside biosynthesis in specific cell types may play in different neurological diseases.

Gangliosides, sialic acid-containing glycosphingolipids, are integral components of cell membranes and participate in a wide variety of biological functions (see [16] and [22] for review). The biosynthesis of gangliosides, including the major brain gangliosides, is a stepwise process dependent upon the expression and intracellular distribution of glycolsyltransferases and sialyltransferases (that add carbohydrate groups and sialic acids, respectively). There are 3 main ganglioside branches but only a- and b-series gangliosides are highly expressed in brain. Due to branch exclusivity, competition between enzymes at key branch points determines the relative expression levels of the final products. Ganglioside biosynthesis is strictly regulated by the activities of glycosyltransferases and is controlled at the level of gene transcription and posttranslational modification, as described in detail previously [23]. GM1/GD1b-synthase is responsible for the synthesis of GM1/GD1b from GM2/GD2 [23] and these products are substrates of the sialyltransferase St3gal2 [21]. One of the roles for GD1a is to serve as a reserve pool for the synthesis of GM1, potentially important for helping maintain GM1 at physiologically relevant levels. Thus, GM1 and GD1a are considered as a functional unit in association with the endogenous membrane-bound sialidase that converts GD1a into GM1[22]. A decrease in expression of *B3GALT4* and *ST3GAL2* genes, as currently observed in residual dopaminergic neurons in the PD SN, is expected to have a major impact on both GM1 and GD1a levels (as well as GD1b and GQ1b levels), reducing expression of these gangliosides, and potentially putting the affected neurons at risk. While GM1 is the ganglioside that has been most studied in relation to PD, due to its putative neuroprotective/neurorestorative properties, it is not clear at this time the extent to which decreased levels of GD1a, GD1b and GQ1b may also contribute to the neurodegeneration of dopaminergic neurons in PD.

Treatment with GM1 following different types of lesions [ex., [24–26]], including in animal models of PD [ex., [27–35], results in significant biochemical and behavioral recovery. In PD models, GM1 administration rescued damaged SN pars compacta dopamine (DA) neurons and increased striatal DA levels in mouse and non-human primate PD animal models [27–35]. Positive effects of GM1 administration on PD animal models has also translated to the clinic, where positive effects of GM1 treatment have been observed in PD patients [36–39].

In our double-blind placebo controlled delayed start study of GM1 in PD [38], we found that GM1 administration had a relatively early appearing symptomatic effect (similar to what we had described in a previous short duration double blind study [36]), however, with long-term use, we showed for the first time clinical evidence that GM1 administration could potentially slow PD symptom progression [38]. This study provided clinical proof-of-concept data, consistent with many pre-clinical findings, that GM1 use could potentially protect dopamine (DA) neurons from dying and/or rescue and restore functionality to damaged DA neurons leading to a lower than expected rate of symptom progression in PD patients. The reasons for the efficacy of GM1 in the clinical studies mentioned above are still not completely understood. However, based on our current findings, we hypothesize that due to downregulation of *B3GALT4* and *ST3GAL2* genes in DAergic neurons, there is decreased expression of GM1 and other major brain gangliosides in PD SN that could increase the vulnerability of these DA neurons to damage. The reasons for these gene expression changes in PD are not known and may represent a fundamental defect leading to the development of PD or may represent a response of these neurons to some toxin or stressor that initiates the disease process. Nonetheless, we suggest that administration of GM1 in the clinical studies mentioned above replaced a critical amount of GM1 lost in the disease and allowed incorporation of sufficient amounts of GM1 into SN DA neurons to enhance functionality and stabilize/protect these neurons.

Recent studies have reported that mice with knockout of the *B4Galnt1* gene have loss of a-and b-series gangliosides including GM1, and develop a progressive parkinsonism [18, 19]. We did not examine *B4GALNT1* gene expression in the current study so we cannot exclude the possibility that there may also be decreased expression of this gene as well. However, since we previously did not observe a decrease in GM2 or GD2 levels in PD SN [20], we do not think that a decrease in *B4GALNT1* gene expression was likely. It has also been reported that there were diminished levels of GM1 and GD1a in occipital cortex from PD brains, suggesting the possibility of a more widespread deficiency in ganglioside expression beyond the SN [19]. Although we did not examine brain regions other than the SN in the current study, we did observe high levels of *B3GALT4* and *ST3GAL2* gene expression in non-DAergic neurons outside of the SN in the PD brain, in the same sections in which decreased gene expression was observed in SN neuromelanin-containing neurons. This observation suggests that the gene expression changes observed in the PD brain may be specific to SN DAergic neurons. The discrepancy between this and the findings reported previously [19] are unclear and require further research to understand.

Currently, the precise role of alterations in ganglioside expression in the degeneration of DAergic neurons in PD is not known, nor is it entirely clear why there is decreased expression of *B3GALT4* and *ST3GAL2* mRNAs in remaining dopaminergic neurons in the PD SN and what this means for the health and survival of these neurons. It is also not known what potential triggers might underlie disrupted ganglioside biosynthesis and expression in PD nor is there any direct information at this time that relates such occurrences to the vulnerability and/or degeneration of DA neurons. However, it is intriguing to hypothesize that intrinsic or acquired defects in ganglioside biosynthesis may lead to altered ganglioside levels which, even with a relatively small reduction in GM1 expression, may contribute to heightened susceptibility of DA neurons to potentially damaging cellular stressors and over time, lead to the neurodegeneration characteristic of PD.

## Conclusions

In summary, residual neuromelanin-containing neurons in the PD SN were found to have decreased expression of the ganglioside biosynthetic genes *B3GALT4* and *ST3GAL2*, consistent with findings of decreased levels of gangliosides GM1, GD1a, GD1b and GT1b in the PD SN [20]. The decreased expression of these genes and the resulting decreases in major brain gangliosides, including GM1, likely increases the vulnerability of these neurons to degeneration in response to a variety of potential stressors. It has been hypothesized that neurodegeneration in PD due to decreased GM1 levels is due at least in part to a failure in trophic signaling (particularly GDNF signaling) [19, 40] and that this GM1-related failure of trophic signaling leads to depressed clearance of and accumulation of alpha-synuclein [19, 40]. Along these lines, recent studies have demonstrated the inhibition of alpha-synuclein aggregation through the interaction of the protein with GM1 [41] and these authors suggested that the presence of GM1 may moderate synuclein-dependent neuronal dysfunction [41]. Together, the current and other findings support an important role of GM1 in the pathogenesis of PD and the continued study of GM1 replacement as an effective therapeutic strategy for the treatment of PD.

## Supporting information

S1 Fig Representative evidence of intact RNA in post mortem samples. In situ hybridization for human Cyclophilin B (*PPIB*), positive control (A) and *dapB*, negative control (B) in a normal case and *PPIB* (C) and *dapB* (D) in situ hybridization in a Parkinson’s disease case. The abundance of red dots in the positive control signifies RNA of sufficient quality for study of the target RNAs. The absence of signal in the negative control demonstrates the specificity of the in situ reaction. Scale = 25 μm.
(TIF)
