# Supplementary figures and images for: Altered expression of genes involved in ganglioside biosynthesis in substantia nigra neurons in Parkinson’s disease
Source: PLoS One. 2018 Jun 14;13(6):e0199189. doi: 10.1371/journal.pone.0199189 (PMC6002063; doi:10.1371/journal.pone.0199189)

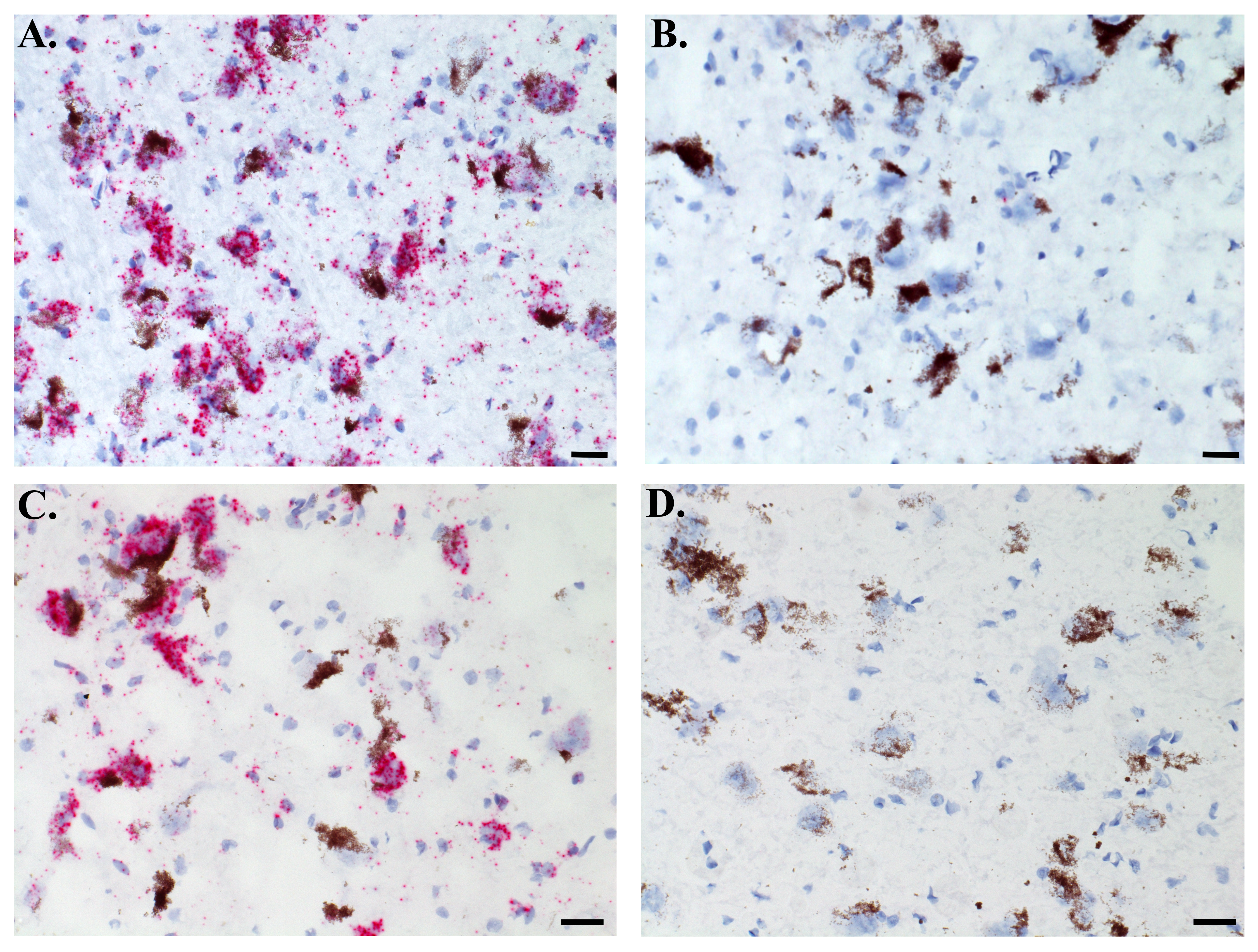

Supplement: S1 Fig — In situ hybridization for human Cyclophilin B (PPIB), positive control (A) and dapB, negative control (B) in a normal case and PPIB (C) and dapB (D) in situ hybridization in a Parkinson’s disease case. The abundance of red dots in the positive control signifies RNA of sufficient quality for study of the target RNAs. The absence of signal in the negative control demonstrates the specificity of the in situ reaction. Scale = 25 μm. (TIF) [file pone.0199189.s001.tif]
